# Immune Response in Severe Infection: Could Life-Saving Drugs Be Potentially Harmful?

**DOI:** 10.1155/2013/961852

**Published:** 2013-09-30

**Authors:** Maja Surbatovic, Jasna Jevdjic, Milic Veljovic, Nada Popovic, Dragan Djordjevic, Sonja Radakovic

**Affiliations:** ^1^Clinic of Anesthesiology and Intensive Therapy, Military Medical Academy, Crnotravska 17, 11000 Belgrade, Serbia; ^2^Faculty of Medicine of the Military Medical Academy, University of Defense, Crnotravska 17, 11000 Belgrade, Serbia; ^3^Clinical Center Kragujevac, Zmaj Jovina 30, 34000 Kragujevac, Serbia; ^4^Faculty of Medical Sciences, University of Kragujevac, Svetozara Markovica 69, 34000 Kragujevac, Serbia; ^5^Institute for Infectious and Tropical Diseases, Intensive Care Unit, Clinical Center of Serbia, Pasterova 2, 11000 Belgrade, Serbia; ^6^School of Medicine, University of Belgrade, Dr Subotica 8, 11000 Belgrade, Serbia; ^7^Sector of Preventive Medicine, Military Medical Academy, Crnotravska 17, 11000 Belgrade, Serbia

## Abstract

Critically ill patients suffer a high rate of nosocomial infection with secondary sepsis being a common cause of death. Usage of antibiotics and catecholamines is often necessary, but it can compromise complex immune response to infection. This review explores influence of these life-saving drugs on host immune response to severe infection.

## 1. Complex Immune Response in Critically Ill Patients with Severe Infection

Severe sepsis and/or trauma complicated with multiple organ dysfunction syndrome (MODS) are leading causes of death in intensive therapy units with mortality rate exceeding 50%. Outcome is not determined only by infection but also by intensity of immunoinflammatory response, which is essential for host defense but if it is uncontrolled it can lead to the MODS [1].

The primary host response to the invading microorganisms will be initiated by resident macrophages and polymorphonuclear cells (PMCs) that are responsible for the primary phagocytosis and subsequent activation and recruitment of polymorphonuclear granulocytes and monocytes. Monocytes will rapidly differentiate, increasing the macrophage population. Various soluble and membrane-bound factors mediate the concerted actions, which constitute the innate response to infections and tissue damage. Cytokines are potent, low molecular weight proteins produced by nucleated cells, particularly those of the immune system, which exert control over the duration and amplitude of the immune/inflammatory response. They have a central role in positive and negative regulation of immune responses and in integrating these reactions with other physiological systems such as the complement and hematopoietic systems. The capacity of cytokines to activate diverse cell types and to incite equally diverse responses underscores the pleiotropism of these inflammatory mediators. There is also significant overlap in bioactivity among different cytokines. Because the effect of cytokines in vivo varies depending on time and location, they can be classified into proinflammatory (T helper, Th1), anti-inflammatory Th2 cytokines and Th17, different from both Th1 and Th2. Many are proinflammatory, for example, tumor necrosis factor- (TNF-) *α*, interleukin- (IL-) 1, IL-8, and high-mobility group box- (HMGB-) 1, others are anti-inflammatory, for example, IL-10 and IL-1 receptor antagonist (ra) and some possess both activities, for example, IL-6. Cytokines act by binding to specific receptors at the target cell membrane, setting off a cascade that leads to induction, enhancement, or inhibition of number of cytokine-regulated genes in the nucleus, thereby modulating the immunological activity of the cell [2–4]. 

Circulating levels of both proinflammatory and anti-inflammatory cytokines are frequently elevated in sepsis, and some relate to increased mortality. They activate numerous cellular processes and other inflammatory mediators that contribute to organ dysfunction. So, patients with severe infection often develop MODS with high morbidity and mortality rate. When microorganisms bind to phagocytic cells through surface TOLL-like receptors (TLRs), so called because they are homologues of the Drosophila protein Toll, a series of intracellular events is initiated that results in the release of cytokines. There are several forms of these receptors with TLR-2 preferentially recognizing Gram-positive bacterial toxins, for example, peptidoglycans and lipoteichoic acid and TLR-4 binding to the lipopolysaccharide (LPS) of Gram-negative bacteria [5–7]. TLRs may recognize either pathogens or endogenous danger signals released by stressed or damaged cell and consequently alert the host by activating the innate immune system. Some molecular fragments from pathogens, such as LPS and bacterial DNA may induce an immune response and are known as specific patterns called pathogen associated molecular patterns (PAMPs). These patterns are recognized by cellular receptors termed pattern recognition receptors (PRRs). Besides PAMPs, there are also several endogenous molecules, such as high-mobility group box- (HMGB-) 1, hyaluronan, and heat-shock proteins (HSPs) that are also able to trigger the immune response through PRRs. These signals are normal cell constituents, which may be released either passively (by necrotic cells), or actively (by stressed cell, in response to cellular injury). Endogenous analogues of PAMPs are called alarmins. These endogenous alarmins and exogenous PAMPs represent two subgroups of the larger category of danger signals termed damage associated molecular patterns (DAMPs) [8]. The explanation of SIRS in absence of obvious microbial infection was provided by Matzinger [9], thus elucidating host response with DAMPs that can activate innate immunity through, among others, TLRs. In addition, a new field for investigation of danger sensing and transmission is opened by the discovery of mitochondrial DAMPs, which activate immune response after cellular disruption by mimicking bacterial infection [10]. The important role of mitochondria in activation of innate immunity is supported by the fact that they contain constituents of their bacterial ancestors which are potentially immunogenic [11]. Interactions between infecting microorganisms and host response can lead to severe sepsis and septic shock. In response to pathogen adherence to an epithelial surface, the host initiates specific mucosal defense mechanisms, in order to prevent microbial invasion. The critical bacterial density needed to initiate an infection is called quorum. Bacterial cell-to-cell communication enables them to assess their population density and interact with the host as a population (quorum-sensing systems). Early nonspecific response system, innate immunity, and more pathogen-specific response system, adaptive immunity, are parts of immune system as a whole [12]. Regardless of the actual underlying cause of severe infection (severe acute pancreatitis, secondary peritonitis, and sepsis secondary to trauma), systemic inflammation can be initiated [13].

The inflammatory response contributes significantly to the morbidity and mortality of critically ill patients and displays high level of interindividual variation. There is tremendous variability seen in the clinical profile and outcome in patients who encounter similar infection as an insult. Genetic polymorphisms in the immune response to infection are associated with the sensitivity to certain infection and with clinical outcomes [14]. Despite significant advances in understanding of the biology of inflammation, improvements in clinical outcomes have been more sporadic and, with few notable exceptions, are related to improvements in supportive care rather than to specific therapies. As a result, in severe infection morbidity, mortality, and cost of treatment remain high.

So, bacterial sepsis is associated with the activation of immune cells by whole bacteria and by bacterial derived products resulting in a local and systemic inflammation. Inflammatory response differs from organ to organ and from organ to peripheral blood, leading to the concept of compartmentalization. The most striking differences exist between tissues and the blood compartment. During infection, live bacteria and whole bacteria killed following the action of complement, defensins, antimicrobial peptides, or antibiotics interact with immune cells. While anti-inflammatory mediators predominate within the blood stream to avoid igniting new inflammatory foci, their presence within tissues may not always be sufficient to prevent the initiation of deleterious proinflammatory response in the different compartments. In severe infection, cytokines are produced in excess and are, therefore, detectable in blood, where they are normally absent. However, the circulating cytokines are merely the tip of the iceberg, and leukocyte-associated cytokines can be identified even when amounts in plasma are undetectable. For a long time, sepsis has been considered to be the result of an overwhelming production of proinflammatory mediators within blood compartment. Presence of circulating cytokines within the blood stream may not always be associated with the maintenance of the proinflammatory process; it may deactivate leukocytes from a further migration within tissues in response to local gradients of chemokines. So both proinflammatory and an opposing anti-inflammatory response occur concomitantly in sepsis [15]. 

Real cause of death and organ failure in most patients dying of sepsis is unknown. Postmortem study results have shown a relative paucity of cell death in most major organs in patients who died of sepsis [16]. One theory is that much of the organ dysfunction in sepsis might be a result of a so-called cellular hibernation response [17, 18]. In most recent review, Hotchkiss with coauthors delineated three potential inflammatory responses in sepsis [19]. Immune responses in sepsis are determined by many factors including pathogen virulence, size of bacterial inoculum, and comorbidities. In the first scenario, although both proinflammatory and anti-inflammatory responses begin rapidly after sepsis, the initial response in patients with severe sepsis, who were previously healthy, is typified by an overwhelming hyperinflammatory, proinflammatory phase with fever, hyperdynamic circulation, and shock. Deaths in this early phase of sepsis are generally due to cardiovascular collapse, metabolic derangements, and multiple organ dysfunction. Although no particular anti-inflammatory therapies have improved survival in large phase 3 trials, short acting anti-inflammatory or anticytokine therapies offer a theoretical benefit. In the second scenario, according to Hotchkiss, many patients who develop sepsis are elderly with numerous comorbidities that impair immune response. When these individuals develop sepsis, a blunted or absent hyperinflammatory phase is common, and patients rapidly develop impaired immunity and an anti-inflammatory state. Immunoadjuvant therapy that boosts immunity offers promise in this setting. And, finally, third theoretical immunological response to sepsis is characterized by cycling between hyperinflammatory and hypoinflammatory states. According to this theory, patients who develop sepsis have an initial hyperinflammatory response followed by hypoinflammatory state. With the development of a new secondary infection, patients have repeat hyperinflammatory response and may either recover or reenter the hypoinflammatory phase. Patients can die in either state. The longer the sepsis continues the more likely a patient is to develop profound immunosuppression. Autopsy results show that most patients admitted to intensive care units (ICUs) for treatment of sepsis had unresolved septic foci at postmortem, suggesting that patients were unable to eradicate invading pathogens and were more susceptible to nosocomial organisms, or both. 

Critically ill patients suffer a high rate of nosocomial infection with secondary sepsis being a common cause of death. This high prevalence of secondary infections argues for the influence of an immune suppression that may, at first glance, appear paradoxical in light of the proinflammatory nature of many critical illnesses. Among patients requiring organ support in ICU, the prevalence of nosocomial infection rises to 25–40% [20]. There is evidence accumulating for the role of proinflammatory mediators in driving immune dysfunction. This may, in part, explain the apparent paradox of immune suppression occurring in a patient with manifestations of hyperinflammation [21]. Clinically, many patients show signs of persisting inflammation and immune-mediated organ damage while simultaneously remaining highly susceptible to secondary infections, suggesting the term complex immune dysfunction syndrome (CIDS) [22]. Immune hypoactivity has now been demonstrated in virtually all immune cell types, including innate actors such as neutrophils, monocytes, tissue macrophages, and dendritic cells, as well as in the adaptive immune system in T cells, B cells, and natural killer (NK) cells. Neutrophils, among other cells, display apparent dualistic state by demonstrating features of both activation and dysfunction simultaneously. Organ dysfunction in critically ill patients is, to a considerable degree, driven by neutrophils [23]. These key immune cells tend to display surface markers of activation, notably elevated levels of CD11b and CD64, but also exhibit profound impairment of phagocytic ability and production of reactive oxygen species (ROS). This apparently paradoxical superposition of both proinflammatory activation and failure of key antimicrobial functions within the same cell type was illuminated by the finding that dysfunction was driven by an excess of the proinflammatory complement split product, anaphylatoxin, C5a [24, 25]. 

New immunoinflammatory paradigm is developed in critically ill trauma patients [26]. The current paradigm explains complications of severe injury as a result of excessive proinflammatory response (systemic inflammatory response syndrome (SIRS) followed temporally by compensatory anti-inflammatory response syndrome (CARS). SIRS represents excessive innate immune response, and CARS represents suppressive adaptive immune response. A second-hit phenomenon results from sequential insults, which leads to more severe, recurrent SIRS and organ dysfunction. The proposed new paradigm involves simultaneous and rapid induction of innate (both proinflammatory and anti-inflammatory genes) and suppression of adaptive immunity genes. Complicated recoveries are delayed, resulting in a prolonged, dysregulated immunoinflammatory state. 

## 2. Antibiotics and Immune Response in Critically Ill Patients with Severe Infection

As far as timing of antibiotic therapy in critically ill patients with severe infection is concerned, facts are rather straightforward and international guidelines strongly recommend initiation of effective intravenous antimicrobials within the first hour of recognition of severe sepsis or septic shock (grade 1B) [27]. Severe sepsis is defined as sepsis-induced tissue hypoperfusion or organ dysfunction with hyperlactatemia. Rationale is as follows: in the presence of septic shock, each hour delay in achieving administration of effective antibiotics is associated with a measurable increase in mortality in a number of studies. Overall, the preponderance of data support giving antibiotics as soon as possible in these patients.

Pivotal research in timing of antibiotic administration in patients with sepsis and septic shock was published by Kumar et al. seven years ago [28]. It was retrospective review of three-patient cohorts of adult septic shock patients. In total, 2731 cases from 14 ICUs were determined to fit the diagnostic criteria for septic shock. Every hour of effective antibiotic delay was associated with approximately 12% (depending on subgroup of patients, mean decrease in survival was 7.6%) decreased probability of survival compared with the previous hour. Overall mortality rate was 56,2%. Survival was similar whether the infection was documented or suspected, whether a plausible pathogen was identified or not, and whether bacteremia was present or absent. In 2154 patients who received effective antimicrobials only after onset of hypotension, mortality rate was 58%. But there was subgroup of 558 patients who received effective antimicrobial therapy before onset of hypotension with mortality rate of 47,8%. One would expect that this subgroup should have lower mortality rate; according to this study similar mortality rate occurred in patients who received effective antimicrobial 5 hours after onset of hypotension and in patients who received effective antimicrobial therapy before onset of hypotension. Also, delay exceeding 36 h increased the risk of death 100-fold with less than 5% surviving. Clearly, these data are surprising giving that bacterial culture and susceptibility results are often not available until after 36 h, not infrequently prompting a belated change of antibiotics, and that many such patients do survive [29]. One interesting research regarding timeliness of appropriate antibiotic therapy and outcome in septic shock patients was published last year [30]. Authors examined the factors responsible for death among bacteremic septic shock patients deemed to have received timely, appropriate antibiotic therapy. Four hundred thirty-six bacteremic patients with septic shock were identified over a 5-year period in single center retrospective study. Though all patients received timely and appropriate antimicrobial therapy, more than half (51,4%) of the patients in the cohort died. Also, more than half (52,3%) of the patients developed septic shock following a hospital-acquired infection. Increasing Acute Physiology and Chronic Health Evaluation II scores and infections acquired in the ICU were independent risk factors for death. Timeliness of antibiotic therapy was not. In his comment [31], Kumar pointed out, among other things, that the fact that authors were unable to identify a relationship between timing of antibiotics and outcomes may, in his opinion, simply reflect how timeliness of antibiotics was defined between studies. In study conducted by Labelle and coauthors, timeliness of administration was linked to time of blood culture draws. Kumar suggests in his comment that this index point may have little relationship to physiological processes like hypotension onset, which have been used as index point in his study.

A systematic review published in 2007 highlighted 21 of 49 reported studies in bacteremic patients that failed to detect any association between inappropriate antibiotic prescription and mortality [32]. The authors were highly critical of the methodologies used to assess whether true differences actually existed or whether unrecognized sources of confounding or biases affected the observations and conclusions, for example, determination as to whether mortality is attributable or not to the infection. They concluded that, without adequately designed research studies in this area, there is little evidence for or against recommendations regarding aggressive empiric therapy with broad-spectrum antibiotics. 

There is one significant factor that influences antibiotic efficacy and severity of illness. Study investigating the interaction between disease severity and efficacy of antibiotic therapy in 142 critically ill patients with ventilator-associated pneumonia showed that inadequate empirical therapy was associated with a poor prognosis only in patients with moderate severity of illness. Conversely, for the group of patients who were most severely ill, neither the adequacy of initial therapy nor the duration of inadequate therapy influenced survival [33]. It seems that detrimental effects of inadequate antibiotic therapy become weaker in the most severely ill patients with short life expectancies [34]. In BASIC study [35] this notion is confirmed. A logistic regression analysis, that was performed on data prospectively collected on 1702 bacteremic ICU patients in 132 ICUs from 26 countries, found that age, illness severity, and immunosuppression were independent predictors for mortality. However, no variable associated with antibiotic policy was significantly associated with death. If the maximum severity of the bacteremic illness was removed from the model, effective first-line antibiotic therapy did reduce mortality, only when started early as empirical treatment (odds ratio 0.58; 95% confidence interval 0.39–0.87). The benefit would thus appear to be derived from early treatment only when commenced before the patient becomes critically ill.

Association between timing of antibiotic administration and mortality from septic shock was focus of yet another research [36] designed to perform preplanned analysis of a multicenter (three urban U.S. emergency departments) randomized controlled trial of early sepsis resuscitation. Data on patients who received an initial dose of antibiotics after presentation to the emergency department were categorized based on both time from triage and time from shock recognition to initiation of antibiotics. The primary outcome was in-hospital mortality. Of 291 included patients, mortality did not change with hourly delays in antibiotic administration up to 6 hours after triage: 1 hour (odds ratio—OR 1.2; 0.6–2.5), 2 hours (OR, 0.71; 0.4–1.3), and 3 hours (OR, 0.59; 0.3–1.3). Mortality was significantly increased in patients who received initial antibiotics after shock recognition compared with before shock recognition (OR, 2.4; 1.1–4.5); however, among patients who received antibiotics after shock recognition, mortality did not change with hourly delays in antibiotic administration. 

A few years ago, Mervyn Singer published paper titled “Treating critical illness: the importance of first doing no harm.” From this provocative and interesting paper, there are several important messages [37]. For example, more than half the 50-plus recommendations made in the surviving sepsis guidelines, which have been endorsed and promoted worldwide, were based solely on expert opinion. Many of the other, more highly graded recommendations relied upon studies with small patient numbers and/or methodological flaws. There are only several Grade A recommendations, supported by at least two large, randomized trials with clear-cut results. The major advances of critical care medicine in the last 20 years have been related more to the recognition and removal of harmful practices rather than to any novel pharmacological or mechanical interventions. Singer states that it is reasonable to question how many currently fashionable strategies may actually prove injurious when submitted to critical examination. 

Over a billion years ago, a bacterium containing the oxygen-consuming respiratory chain is likely to have invaded the early eukaryotic cell. Most of the bacterial genetic information was subsequently transferred to the nucleus, transforming these bacterial symbionts into “slave” mitochondrial organelles. This provided a far more efficient system for using available energy sources and also protected the cell against the potentially toxic effects of oxygen. More than 90% of total body oxygen consumption is used to generate adenosine triphosphate (ATP) by the mitochondrial electron transport chain, and this, in turn, provides more than 90% of the energy. One of the hypothesis, made by Singer, to explain the pathophysiology of MODS following infection and other inflammatory insults is a mitochondrial shutdown leading to “energy failure” and a consequent inability to drive the various metabolic processes that maintain normal cellular functioning. Inflammatory mediators released in considerable excess in sepsis, such TNF-alpha and nitric oxide (NO), are known to directly inhibit mitochondrial respiration [38–41]. If the cell attempts to continue to function normally despite inadequate energy production, the resulting fall in adenosine triphosphate will trigger necrotic and apoptotic death pathways. However, as this process is not immediate (it takes hours to days to develop fully), the cell has time to potentially adapt to this prolonged, life-threatening insult. It is likely to do so by entering a hibernation-like state. The impressive and almost total absence of cell death seen in organs that have “failed” biochemically and/or physiologically [42] lends credence to this hypothesis. Restoration of cellular function and thus recovery from organ failure must therefore depend upon repair of damaged mitochondria and/or production of new organelles, a process known as mitochondrial biogenesis. Carre with coauthors conducted research regarding association between survival in critical illness and mitochondrial biogenesis [43]. Muscle biopsies were taken from 16 critically ill patients recently admitted ICU (average 1-2 days) and from 10 healthy, age-matched patients undergoing elective hip surgery. Survival, mitochondrial morphology, mitochondrial protein content and enzyme activity, mitochondrial biogenesis factor mRNA, microarray analysis, and phosphorylated (energy) metabolites were determined. Eventual survivors responded early to critical illness with mitochondrial biogenesis and antioxidant defense responses. Authors concluded that these responses may partially counteract mitochondrial protein depletion, helping to maintain functionality and energetic status. Impaired responses, as suggested in nonsurvivors, could increase susceptibility to mitochondrial damage and cellular energetic failure or impede the ability to recover normal function. 

Antibiotics are used to fight bacteria. Many of the antibiotic classes, such as the penicillins and cephalosporins, are bactericidal through cell-wall disruption, whereas other classes, such as chloramphenicol and aminoglycosides, act in a bacteriostatic manner by inhibiting protein synthesis. However, by virtue of their action, the cell-wall disrupters, in particular the cephalosporins, cause increased levels of endotoxin release from Gram-negative bacteria [44] and lipoteichoic acid and peptidoglycan release from Gram-positive bacteria [45]. This enhanced toxin release leads to significantly higher inflammatory mediator production. This may well explain the rapid clinical deterioration often seen in patients with sepsis after the first dose of cidal antibiotics, and, for that, at least in part, Jarisch-Herxheimer reaction is responsible. The Jarisch-Herxheimer reaction (JHR) is a classic clinical syndrome in which there is a profound worsening of symptoms immediately following antimicrobial treatment of infection. JHR was first described at the turn of the last century in two independent papers by Jarisch in Vienna and Herxheimer in Berlin, in syphilitic patients following treatment with mercury. It is now recognized that similar transient phenomena occur soon after the first dose of an appropriate antibiotic in the treatment of wide spectrum of infectious diseases. The JHR in these conditions is classically associated with an increase in body temperature of approximately 1–1.5°C within 1 to 2 hours of antibiotic administration, rigors, a fall in systemic arterial blood pressure, and a fall in peripheral blood white cell count. A decade and a half ago, detailed analysis of cytokine release in the early phase of JHR showed that TNF concentration increases within 30 min following penicillin administration and that this increase preceded pyrexia and the rises in plasma IL-6 and IL-8 concentrations. In summary, TNF appeared in the plasma before the onset of symptoms, IL-6 was detected as symptoms developed, and IL-8 was detected in plasma well after the onset of rigor and pyrexia. Finally, the plasma concentrations of IL-8 recorded in JHR were ten times higher than those recorded following bolus endotoxin administration [46]. A delayed and potentially significant effect of antibiotics may be seen through their inhibition of mitochondrial activity and biogenesis (mitochondria are genetically linked to bacteria). Two recently published studies have used proinflammatory blood marker procalcitonin to guide discontinuation of antibiotics. Results from PRORATA trial [47] showed that mortality of patients in the procalcitonin group seemed to be noninferior to those in the control group at day 28 and at day 60. Patients in the procalcitonin group had significantly more days without antibiotics than did those in the control group (14.3 days versus 11.6 days, but use of procalcitonin to guide antibiotic therapy did not change mortality). In the second trial [48], a total of 1200 critically ill patients were included. The primary end point was death from any cause at day 28; this occurred for 31.5% patients in the procalcitonin arm and for 32.0% patients in the standard-of-care-only arm. Length of stay in the intensive care unit was increased by one day (*P* = .004) in the procalcitonin arm, the rate of mechanical ventilation per day in the intensive care unit increased 4.9%, and the relative risk of days with estimated glomerular filtration rate <60 mL/min/1.73 m was 1.21. Authors concluded that procalcitonin-guided antimicrobial escalation in the intensive care unit did not improve survival and did lead to organ-related harm and prolonged admission to the intensive care unit. The procalcitonin strategy like the one used in this trial cannot be recommended. Results from these two large studies did not confirm the use of procalcitonin as a “gold standard” in antibiotic stewardship. Ongoing inflammation is far too complex to be direct surrogate of ongoing bacterial activity. Shorter duration of antibiotic therapy is preferable [49].

Antibiotics, as other drugs, have obvious side-effects: rashes, liver and renal dysfunction, and so forth. Overgrowth of multidrug-resistant bacteria and fungi can occur, as well as Jarisch-Herxheimer reaction to release of bacterial products. Antibiotics are immunomodulatory and can compromise mitochondrial function. Main body of evidence shows that benefit of antibiotics is before the patient gets very ill, which suggests that microbe itself is less important later in disease process after patient becomes ill. In that stage immunoinflammatory response is crucial and often detrimental to the patient. 

## 3. Catecholamines and Immune Response in Critically Ill Patients with Severe Infection

Severe infection and sepsis result not only in immune activation but also in activation of number of other neurohumoral systems, with the catecholamines being key mediators of the frequently seen tachycardia and hyperdynamic circulation. Exogenous catecholamines and adrenergic drugs are regularly administered to the patients to reverse vasodilatation and later stage reductions in cardiac output [22]. Acting via beta-receptors, these drugs can impair functions of neutrophils and T cells, and, at least in part, immune suppression seen in sepsis is beta-adrenergic mediated. Catecholamines have many effects distant from their cardiovascular actions. They have metabolic effects including increased beta-oxidation of fats; they are proarrhythmogenic; they have proinflammatory and anti-inflammatory effects, and they can alter both immunity and mitochondrial function [37, 50–53]. 

Lyte and coauthors [54] focused their research on hypothesis that administration of inotropic agents via indwelling intravenous catheters may stimulate growth and formation of biofilms by *Staphylococcus epidermidis*. Inocula representing physiologically relevant infecting doses of *Staphylococcus epidermidis* were incubated in a minimum medium supplemented with fresh human plasma in the presence or absence of pharmacological concentrations of noradrenaline or dobutamine. Biofilm formation on polystyrene and medical-grade silicone was examined. After incubation, cultures were assessed for growth and formation of biofilms by colony counting and scanning electron microscopy. The production of exopolysaccharide, a major constituent of *Staphylococcus epidermidis* biofilms, was also assessed by use of immunofluorescence microscopy. Authors found that incubation of *Staphylococcus epidermidis* with catecholamine inotropes in the presence of human plasma resulted in a significant increase in growth compared with control on both polystyrene and silicone surfaces, with pronounced increases in biofilm formation as visualized by scanning electron microscopy. Authors concluded that the ability of catecholamine inotropic drugs to stimulate bacterial proliferation and biofilm formation may be an etiological factor in the development of intravascular catheter colonization and catheter-related infection. The removal of iron from transferrin for subsequent use by *Staphylococcus epidermidis* is a possible mechanism by which catecholamine inotropes stimulate bacterial growth as biofilms. Another interesting research from the same group of authors focused on growth stimulation of intestinal commensal *Escherichia coli* by catecholamines, especially in trauma-induced sepsis [55]. Trauma is well recognized to result in the immediate and sustained release of stress-related neurochemicals such as catecholamine noradrenaline. In addition to their ability to function as neurotransmitters, catecholamines can also directly stimulate the growth of a number of pathogenic bacteria. The development of trauma-associated sepsis has often been linked to the ability of otherwise normal commensal bacteria to invade and penetrate the gut mucosal barrier. The aim of this research was to examine whether catecholamines could also stimulate the growth of commensal *Escherichia coli* strains of the type present in the intestinal tract at the time of a traumatic event. Authors found that the growth of a range of nonpathogenic isolates of *Escherichia coli* of human and environmental origin was significantly increased in the presence of catecholamines. A primary mechanism by which catecholamines increase bacterial growth was shown to be iron removal from lactoferrin and transferrin and subsequent acquisition by bacteria. The 3,4-dihydroxybenzoyl (catechol) structure of the catecholamines was further demonstrated to be critical to iron acquisition. The synthetic catecholamine inotropes dobutamine and isoprenaline, as well as noradrenaline metabolites that retained the catechol structure, were also active, whereas noradrenaline metabolites in which the catechol moiety had been modified were not. Authors concluded that there is a role for catecholamine-mediated bacterial iron supply in the pathophysiology of gut-derived sepsis due to trauma. 

Furthermore, host resistance to bacteria might be compromised because both catecholamines and dopaminergic agents, such as dopamine, dobutamine, and dopexamine, affect activity and survival of most, if not all, immune-cell populations [56]. Adrenaline and noradrenaline decrease the proinflammatory effect of endotoxin but enhance production of the anti-inflammatory interleukin-10. This increase in interleukin-10 contributes to an immunosuppressive effect on monocytes and macrophages. Noradrenaline also has a direct inhibitory effect on the energy metabolism of monocytes and macrophages [57]. 

Plasma catecholamine concentrations rise up to 20-fold in critical illness. Concentrations return to normal over 5 days in eventual survivors but rise still further in nonsurvivors, many of whom receive exogenous catecholamines. Better alternatives to catecholamines are needed, which might include agents as diverse as vasopressin, levosimendan, or specific inducible inhibitors of nitric oxide synthase. Better definition of lowest acceptable blood pressure in individual patients is also needed to minimize the harmful effects of excessive catecholamine dosing. Additionally, the use of concurrent medications that contribute to hypotension (e.g., excess sedative dosing) and vascular hyporeactivity (e.g., etomidate) should be limited [58]. Guidelines recommend mean blood pressure values ≥65 mmHg for patients with severe sepsis, but with no firm scientific basis. However, data from septic shock studies indicate that these pressure targets are regularly exceeded, often by ≥20 mmHg. Higher mortality was noted in those where higher mean blood pressure values were generated using progressively higher catecholamine doses. Overadjustment of blood pressure and unnecessary vasopressor use over adjustment of blood pressure and unnecessary vasopressor use may thus be harmful [59].

Because septic patients are often vasodilated and hypotensive, beta-blockers are traditionally not used. But, Ackland with co-authors explored in animal model (male adult Wistar rats) the hypothesis that beta-1 adrenoceptor blockade may be protective through the attenuation of sympathetic hyperactivity and catecholaminergic inflammatory effects on cardiac and hepatic function [60]. Interventions consisted of peripheral beta 1-adrenoceptor blockade through daily intraperitoneal injection (metoprolol, atenolol) or central nervous system beta 1-adrenoceptor blockade (intracerebroventricular metoprolol) to achieve approximately 20% heart rate reduction in rats for 2 days before or after the induction of lethal endotoxemia, cecal ligation and puncture, or fecal peritonitis. Authors found that peripheral beta 1-adrenoceptor blockade established for 2 days before lethal endotoxemia markedly improved survival in both metoprolol and atenolol-treated rats. Metoprolol pretreatment reduced hepatic expression of proinflammatory cytokines and lowered plasma interleukin-6 levels. Myocardial protein expression of interleukin-18 and monocyte chemoattractant protein-1, key mediator of cardiac dysfunction in sepsis, were also reduced. Peripheral beta 1-adrenoceptor blockade commenced 6 hours after lethal endotoxemia or fecal peritonitis did not improve survival. However, arterial blood pressure was preserved and left ventricular contractility restored similar to that found in nonseptic controls.

Beta-blockers can prevent downregulation of adrenergic receptors, thus preserving cardiac function and improving outcomes [61]. Several studies showed that in septic patients beta-blockade compromised neither oxygen utilization, ATP availability, nor the macrocirculation [62, 63]. Jeschke with coauthors focused their research to determine the effect of propranolol administration on infection, sepsis, and inflammation in severely burned pediatric patients [64]. Study showed that propranolol significantly decreased resting energy expenditure (REE), which is used to determine hypermetabolic response. Thirty percent of patients in control group developed infections compared to 21% in propranolol group. The incidence of sepsis was 10% for controls and 7% for propranolol. Analysis of the cytokine expression profile in 20 patients in each group revealed that propranolol significantly decreased serum tumor necrosis factor and interleukin-1 beta compared with controls. Authors concluded that propranolol treatment attenuates hypermetabolism and does not cause increased incidence of infection and sepsis.

These concepts tie in well with the paradigm of allostasis [65] and its applicability to critical care. Allostasis is an adaptive phenomenon whereby the body adjusts and adapts itself to various stressors (e.g., exercise, emotion, and hunger) to maintain homeostasis in systems essential for life. More stress will increase allostatic load, placing greater pressure on these adaptive systems. Severe and/or prolonged stress can result in allostatic overload, a decompensation with pathological effects on various systems (immune, hormonal, metabolic, cardiovascular, and gut). 
